# Psychological, social, and behavioral barriers and facilitators to prehabilitation before cancer surgery: a qualitative systematic review

**DOI:** 10.3389/fonc.2026.1903631

**Published:** 2026-09-17

**Authors:** Hongxia Xu, Yihong Xu, Gongjie Shi, Qiulei Ding, Xiao Liang, Hongying Pan

**Affiliations:** 1 Department of Nursing, Sir Run Run Shaw Hospital, School of Medicine, Zhejiang University, Hangzhou, China; 2 Department of General Surgery, Sir Run Run Shaw Hospital, School of Medicine, Zhejiang University, Hangzhou, China

**Keywords:** barriers and facilitators, cancer surgery, prehabilitation, psychological and behavioral determinants, psycho-oncology, qualitative systematic review

## Abstract

**Background:**

Prehabilitation before cancer surgery improves postoperative outcomes, yet patient participation remains suboptimal. This qualitative systematic review aimed to synthesize psychological, social, and behavioral barriers and facilitators to preoperative prehabilitation among adult cancer patients.

**Methods:**

A systematic search was conducted in CNKI, Wanfang Data, CBM, VIP, PubMed, Web of Science, The Cochrane Library, Embase, and CINAHL from inception to January 13, 2025, with an updated search on May 31, 2025. A total of 1,184 articles were screened, and 20 qualitative studies were included. Quality appraisal was performed using the Critical Appraisal Skills Programme (CASP). Thematic analysis was employed for data synthesis.

**Results:**

The analysis identified 7 facilitating factors: (1) Introduction Mechanisms, (2) Value Recognition, (3) Support Systems, (4) Communication Strategies, (5) Motivation and Feedback Mechanisms, (6) Program Design Optimization, and (7) Process Convenience and Program Accessibility, and 4 barrier factors: (1) Cognitive and Informational Barriers, (2) Individual Factors, (3) Clinical Process Limitations, and (4) Implementation Environment Barriers. When mapped onto a psychological, social, and behavioral framework, psychological facilitators included regained sense of control, mood regulation, and altruistic motivation; psychological barriers encompassed fear, low self-efficacy, and illness-related distress. Social facilitators comprised family involvement, peer support, and professional encouragement; social barriers included insufficient family support and communication gaps. Behavioral facilitators were driven by immediate progress feedback and self-monitoring; behavioral barriers included scheduling conflicts and low exercise confidence.

**Conclusion:**

This synthesis reveals a persistent “cognitive-behavioral gap”—patients recognize the value of prehabilitation yet encounter barriers that prevent them from translating this recognition into sustained action. Addressing this gap requires integrated, multi-level strategies that enhance self-efficacy, mobilize social support, embed behavioral reinforcement mechanisms, and address systemic barriers.

**Systematic review registration:**

https://www.crd.york.ac.uk/prospero/, identifier CRD420250650240.

## 1 Introduction

Cancer patients, particularly those undergoing major oncologic surgery, often face significant challenges during postoperative recovery. Enhanced Recovery After Surgery (ERAS) protocols have been demonstrated to substantially improve postoperative recovery quality (1–3). As the initial phase of ERAS programs (4), prehabilitation plays a pivotal role in optimizing preoperative functional status and enhancing physiological reserve (5–7). Prehabilitation has been increasingly recognized as a potentially important component of perioperative cancer care, with studies reporting associations with improved functional capacity, reduced complications, and shorter hospital stays in selected populations (8, 9).

Contemporary prehabilitation strategies have evolved from unimodal interventions focusing on exercise, psychological, or nutritional aspects (10–13) to comprehensive multimodal programs integrating physical, psychological, and nutritional components (14–16). With technological advancements, prehabilitation has further expanded into remote delivery models (17, 18). However, despite these conceptual innovations, clinical implementation faces numerous challenges, including those associated with remote delivery (19), as well as broader individual and structural barriers (20–23). Importantly, many of these barriers are psychological, social, or behavioral in nature, with the social dimension encompassing both interpersonal relationships and broader organizational and environmental contexts. Conversely, facilitators often operate through psychosocial mechanisms such as regained sense of control, positive feedback, and motivational support. Identifying these facilitators and barriers is crucial for optimizing prehabilitation strategies and improving patient engagement (24).

While previous mixed-methods studies have examined factors influencing prehabilitation in cancer patients or major surgery populations (22, 23), the predominance of quantitative analyses has marginalized qualitative findings, resulting in insufficient exploration of patient experiences, emotional responses, and social contexts. Qualitative research is particularly well-suited to uncover how patients perceive, interpret, and respond to prehabilitation psychologically and socially—yet these insights remain underexploited. Furthermore, although digital health technologies (e.g., remote monitoring and mobile health) present new opportunities for prehabilitation (25), they also introduce novel psychosocial and behavioral factors (19) that remain under investigated.

A qualitative systematic review adopting a psychological, social, and behavioral framing is warranted for three reasons.

First, theoretical frameworks from health psychology and behavioral medicine—such as the COM-B model (26) and the Theoretical Domains Framework (27)—consistently identify psychological constructs (e.g., beliefs about consequences, self-efficacy, emotion), social factors (e.g., social support, social influence), and behavioral regulation mechanisms (e.g., feedback, goal setting, self-monitoring) as core determinants of health behavior engagement. A socio-ecological perspective (28) further extends this understanding by recognizing that these individual and interpersonal determinants are themselves embedded within, and shaped by, broader organizational and environmental contexts. Prehabilitation participation, as a complex health behavior, is likely shaped by these same dimensions.

Second, while existing qualitative syntheses have examined barriers and facilitators to prehabilitation (22, 23, 29), most have either adopted broad, atheoretical categories or relied on framework-driven approaches. A focused psychological, social, and behavioral lens allows us to foreground how patients think, feel, interact, and act in relation to prehabilitation—without being constrained by a single pre-existing framework.

Third, from a clinical perspective, identifying modifiable psychological (e.g., fear, low confidence), social (e.g., lack of family encouragement), structural (e.g., financial burden, limited service accessibility), and behavioral (e.g., absence of self-monitoring) factors provides actionable targets for intervention. Unlike fixed demographic or disease-related barriers, these dimensions can be addressed through psychosocial support, behavioral counseling, and family involvement strategies—aligning directly with the scope of psycho-oncology.

This review was guided by the question: What are the psychological, social, and behavioral barriers and facilitators to preoperative prehabilitation among adult cancer patients? Therefore, this qualitative systematic review aims to systematically synthesize existing qualitative evidence and explicitly examine the psychological, social, and behavioral facilitators and barriers to prehabilitation in adult cancer patients, thereby providing an evidence-based foundation for developing psychosocially informed, behaviorally oriented prehabilitation strategies.

## 2 Materials and methods

### 2.1 Design

The reporting of this study’s findings adheres to the Enhancing Transparency in Reporting the Synthesis of Qualitative Research (ENTREQ) guidelines (30) and the Cochrane Qualitative Review Reporting Guidelines (31). The study protocol was registered with PROSPERO (CRD420250650240).

### 2.2 Search method

A researcher with extensive literature search expertise systematically searched the following databases using a combination of subject headings and free-text terms: China National Knowledge Infrastructure (CNKI), Wanfang Data Knowledge Service Platform, Chinese Biomedical Literature Database (CBM), VIP Database, PubMed, Web of Science, The Cochrane Library, Embase, and CINAHL. The initial search was conducted on January 13, 2025. Prior to manuscript submission, an updated search was conducted on May 31, 2025, covering the period from January 14, 2025, to May 31, 2025, to ensure the synthesis reflected the most current evidence. The updated search identified two additional eligible studies. The detailed search strategies are presented in Appendices S1.

Two independent reviewers screened the titles and abstracts of retrieved citations against the predefined inclusion and exclusion criteria. Any citations that either reviewer deemed uncertain were advanced to full-text review. Subsequently, both reviewers independently assessed the full texts of potentially eligible studies for final inclusion. Discrepancies were resolved through consultation with a third reviewer. The final search results were presented using a PRISMA flow diagram (**Figure 1**).

**Figure 1:**
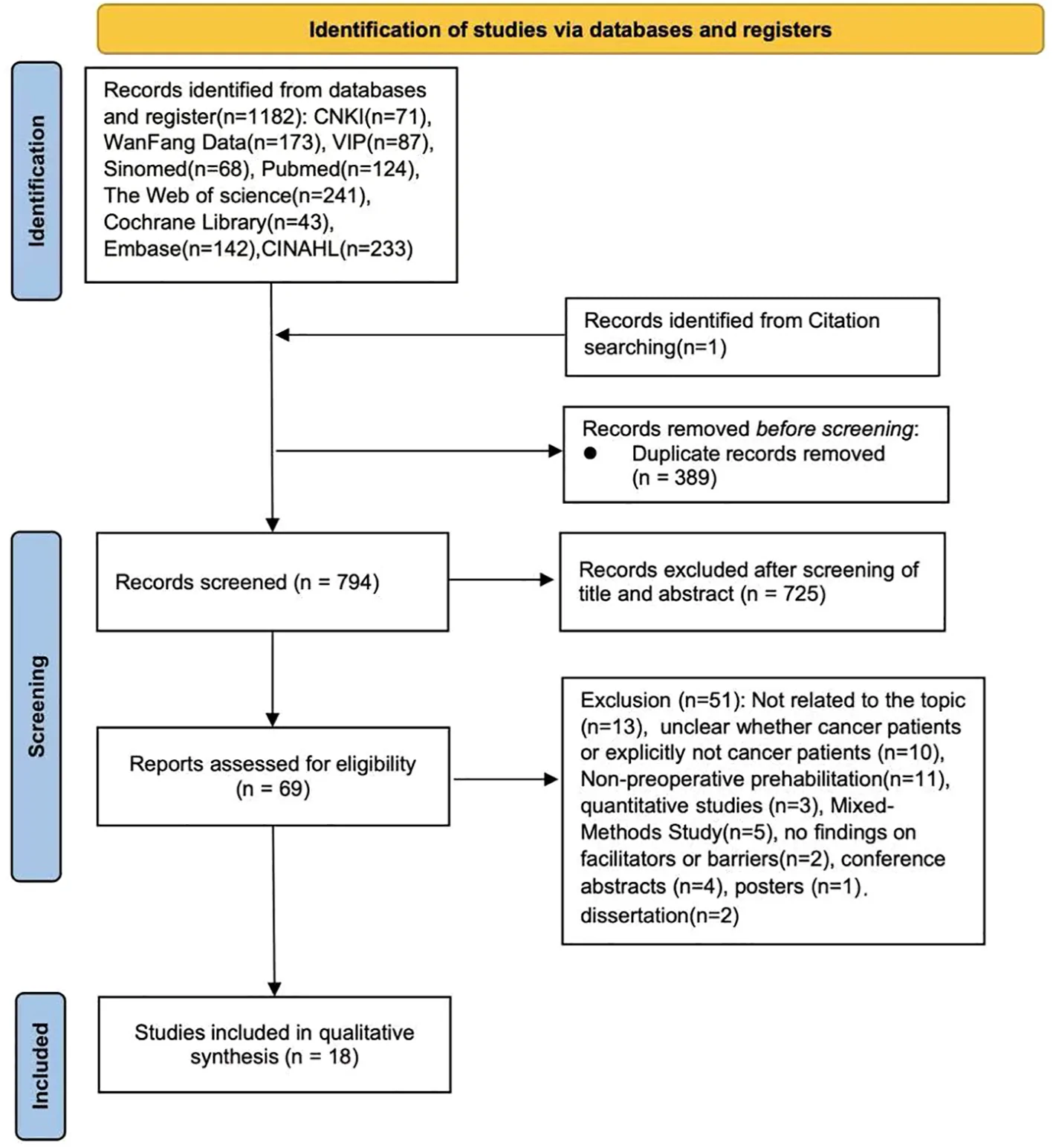
PRISMA flow diagram of the initial study selection process. The initial search (conducted on January 13, 2025) identified 1,182 records. Of these, 18 studies met the inclusion criteria. An updated search (conducted on May 31, 2025) using the same strategies identified two additional eligible studies, bringing the total number of included studies to 20.

The SPICE framework (32) was employed to develop the search strategy, incorporating the following key elements:

(S) Setting: Hospital, community, or home-based

(P) Perspective: Cancer patients, family members of cancer patients, healthcare professionals

(I) Intervention: Prehabilitation

(C) Comparison: Not applicable

(E) Evaluation: Facilitators and barriers

#### 2.2.1 Inclusion and exclusion criteria

Studies were included if they: (1) involved adult patients (≥18 years) scheduled for cancer-related surgery, their family members, or healthcare professionals delivering prehabilitation; (2) examined preoperative prehabilitation interventions encompassing nutritional, psychological, or exercise components; and (3) employed qualitative methodologies, and (4) reported findings related to barriers, facilitators, or factors influencing participation in prehabilitation.

Studies were excluded based on: (1) unavailability of complete data; (2) duplicate publications (only the most comprehensive version retained); (3) non-English or non-Chinese language publications; (4) concurrent investigation of broader perioperative care beyond prehabilitation; (5) mixed patient cohorts where cancer patients could not be analyzed separately; (6) mixed-methods studies with inseparable qualitative components; and (7) thesis or dissertation formats.

### 2.3 Critical appraisal

The quality appraisal was performed with Critical Appraisal Skills Programme (CASP) (33). The methodological quality of the included qualitative studies was rigorously evaluated based on three critical aspects: (1) result validity, (2) result interpretation, and (3) local applicability. This three-dimensional appraisal framework follows established qualitative research assessment principles while maintaining focus on methodological rigor and practical relevance.

The methodological quality assessment for the included studies was independently conducted by two researchers (Reviewer 1 and Reviewer 2), one of whom is an expert in qualitative systematic reviews. Any discrepancies between the two assessors were resolved through consultation with a third independent qualitative research expert (Reviewer 3).

### 2.4 Data extraction

The following information was systematically extracted from each included study and will be presented in **Table 1**: publication year, country, study methodology, participant characteristics (including whether patients were interviewed after completing prehabilitation), interview timing relative to the intervention, sample size, sampling method, data collection procedures, data analysis approach, identified prehabilitation-related themes, and main findings.

**Table 1:** Methodological characteristics of included studies.

Author(Year)	Country	Method	Participants	Participant prehabilitation experience	​Interview timing​	Sample size​	​​Sampling method	Data collection approach	Data analysis method​​	Prehabilitation intervention type
Hogan et al., 2019 (34)	Australia	Qualitative study	Participants had undergone pelvic exenteration	Yes	Postoperative	20	Consecutive sampling	Semi-structured interviews via telephone	Inductive thematic analysis	Oral Nutrition Support (ONS)
Agasi-Idenburg et al., 2020 (35)	Netherlands	Qualitative, observational study	Patients aged 65 years and older,surgically treated for colorectal cancer, informal care givers ,health care providers with experience in prehabilitation	Yes	Both	15,13,9	Purposeful sampling	One-on-one interviews using open-ended questions	Content analysis, thematic analysis, Open & Axial Coding of Grounded thoery	Physical exercise
Karlsson et al., 2020 (36)	Sweden	Qualitative interview study	Persons ≥ 70 years of age scheduled for colorectal cancer surgery	No	Preoperative​​	17	Purposeful sampling	Individual semistructured interviews, face-to-face or by telephone	Inductive content analysis	Physical activity and exercise
Beck et al., 2020 (37)	Denmark	Phenomenology	Colorectal cancer/ovarian cancer patients were scheduled for cytoreductive surgery (CRS) with hyperthermic intraperitoneal chemotherapy (HIPEC) or not	Yes	​​Postoperative	16	Purposeful sampling	Semi-structured, face-to-face, one-on-one interviews	Malterud’s principles of systematic text condensation	Six general recommendations about exercise, nutrition, relaxation, smoking cessation, alcohol cessation, and other preparation
Cooper et al., 2022 (38)	UK	Qualitative study	Participants were adults aged ≥18 years with locally advanced operable oesophagogastric adenocarcinoma due to receive neoadjuvant chemotherapy treatment	Yes	​​Postoperative	22	/	Focus group discussions and individual semi-structured interviews, face-to-face or by telephone or convened online	Thematic analysis	Physical activity and exercise intervention
Heil et al., 2022 (39)	Netherlands	Qualitative study	Healthcare professionals( surgeon, specialized nurse, dietician, physical therapist) involved in preoperative colorectal cancer surgery	Not applicable	Not applicable	13	Purposeful sampling	Semi-structured interviews, face-to-face or by telephone	/	Prehabilitation
van der Zanden et al., 2022 (40)	Netherlands	Qualitative study	Gynecologic oncological patients aged≥60 years and healthcare professionals	/	/	16,20	Convenience sampling and purposive sampling	Semi-structured interviews,face-to-face or using videoconferencing or by telephon	Inductive thematic template analysis	Prehabilitation
Wu et al., 2022 (19)	UK	Descriptive qualitative study	·adults (≥18 years) with a new diagnosis of cancer ·were based in the community (i.e., had been discharged from the hospital following surgery)	Yes	/	22	Purposive sampling	Individual, semistructured interviews	Deductive content analysis;thematic analysis	Tele-Prehabilitation
Barnes et al., 2023 (41)	Canada	Social Constructivist	Older patients (≥ 60 years) having elective cancer surgery, and who were living with frailty (Clinical Frailty Scale ≥ 4)	Yes	/	15	Purposive sampling	Semi-structured and contained open-ended questions informed by the TDF,by telephone	Deductive content analysis	Exercise prehabilitation(aerobic activity, strength and stretching, and nutritional advice)
Paulo et al., 2023 (42)	USA	Exploratory qualitative design	·Being 18 years and older ·Consented to the prehabilitation study ·patients with kidney cancer	Yes	Both	20	Convenience sampling	Semi-structured interviews	Content analysis	Physical activity prehabilitation
Powell et al., 2023 (43)	UK	Qualitative investigation	Adult patient(people with colorectal, lung or oesophago-gastric cancer) and clinician	Both	/	18,24	Purposive sampling	semi-structured phone/ video-call interviews(patient);online questionnaire with free-response and categorical-response questions(clinician)	Thematic analysis; structured using the Framework approach	Exercise, with nutritional status and mental wellbeing also assessed and supported
Voorn et al., 2023 (44)	Netherlands	Qualitative research	Healthcare professionals, patients(Non-small cell lung cancer, NSCLC), and informal caregivers	No	​​Postoperative	12,17,16	/	one-to-one semi-structured interview(healthcare professionals - video consulting online; patients and informal caregivers-face to face)	Thematic analysis	A multimodal prehabilitation program, including physical exercise training, nutritional and psychological support, and/or coaching towards lifestyle changes for patients with operable NSCLC
Wang et al., 2023 (45)	China	Qualitative Study	Older patients(≥ 60 years) with colorectal cancer	Yes	Preoperative​​	12	Purposive sampling	Semi-structured, face-to-face interviews	Directed content analysis	Exercise prehabilitation, including breathing exercises, aerobic exercise, and anaerobic exercise
Casanovas-alvarez et al., 2024 (46)	Spain	Descriptive phenomenological study	Patients with breast cancer undergoing neoadjuvant treatment and awaiting surgery	Yes	/	16	/	Focus groups; face to face	Thematic analysis	Nordic walking training, muscle strengthening exercises and therapeutic education
Clemons et al., 2024 (47)	USA	Qualitative study	Adult patients who had undergone or planned to undergo major abdominal surgery	No	Not applicable	11	Purposive sampling	One-on-one interviews;by phone or face-to-face	Thematic analysis, while including elements of grounded theory methodology and coding reliability	Not specified
Eser et al., 2024 (48)	Swiss	Qualitative research	Patients awaiting lung resection(NSCLC or metastatic disease)	Yes	Both	22	/	Face-to-face interviews	Thematic analysis	Multimodal clinical prehabilitation
Sier et al., 2024 (49)	Netherlands	Phenomenological approach	Underwent surgery for colorectal cancer	Yes		6	/	Semi-structured, individual, face-to-face interviews	Thematical approach	Multimodal Prehabilitation(excecise,nutrition)
Zhang et al., 2024 (50)	China	Descriptive qualitative study	Older patients(≥ 60 years) with early-stage lung cancer	Yes	/	15	Purposive sampling	Semi-structured, face-to-face interviews	Content analysis	Multimodal Prehabilitation(Smoking cessation and alcohol moderation, Aerobic exercise, Resistance training, Respiratory muscle training, Nutritional prehabilitation, Psychological prehabilitation)
Saggu et al., 2025 (51)	UK	Qualitative study	Advanced ovarian cancer patients undergoing or having undergone primary debulking surgery; both prehabilitation-naïve and experienced (MILE programme)	Both	Both (pre and post-operative)	21	Purposive, maximum variation sampling	Semi-structured interviews (face-to-face or by telephone)	Thematic analysis	Multimodal prehabilitation (physical activity, nutrition, psychological support)
Zhang et al., 2025 (52)	China	Qualitative descriptive study	Clinical professionals (clinicians, ward nurses, rehabilitators, psychologists, dietitians, clinical administrators) caring for elderly patients (≥60 years) with early-stage lung cancer	Not applicable (professional perspective)	Not applicable	12	Purposive sampling	Semi-structured interviews (face-to-face)	Content analysis	Multimodal prehabilitation (exercise, breathing, nutrition, psychological support, smoking/alcohol cessation)

“Participant prehabilitation experience” indicates whether interviewed patients had personally undergone the prehabilitation program prior to the interview. “Not applicable” denotes studies involving healthcare professionals as participants, for whom prehabilitation experience does not apply. “Not specified” indicates that the original study did not report this information. or Clemons et al. (2024), the study focused on barriers to prehabilitation participation without specifying a particular intervention type; hence no specific intervention type is listed.

### 2.5 Data synthesis

Thematic analysis (53) was employed to conduct line-by-line analysis of the extracted data. Key sentences or concepts related to prehabilitation facilitators or barriers were annotated and assigned concise descriptive codes. These initial codes were then categorized into groups with similar attributes to form preliminary candidate themes. Subsequently, the research team rigorously examined these candidate themes to ensure internal consistency within each theme and clear demarcation between themes, while eliminating redundant or irrelevant themes. Concurrently, the themes were validated against the original data to verify their reliability and representativeness. Finally, each definitive theme was precisely defined and appropriately named to accurately reflect its conceptual content.

Following the inductive thematic synthesis, the identified themes were further mapped onto psychological, social, and behavioral dimensions. This deductive mapping step was guided by established health behavior frameworks (COM-B model; 26) and served to align the findings with the study’s conceptual framing. Two researchers (Reviewer 1 and Reviewer 2) independently assigned each theme to one or more dimensions, with disagreements resolved through consultation with a third researcher (Reviewer 3). This mapping was performed to complement—not replace—the inductively derived themes and to enhance the clinical interpretability of the findings.

## 3 Results

### 3.1 Characteristics of the included studies

After full-text review and eligibility assessment, 20 studies were included for analysis. Among these, 18 were identified from the initial search (January 13, 2025), and 2 were identified from the updated search (May 31, 2025). The basic characteristics of the included studies are presented in **Table 1**, while their main findings can be found in **Supplementary Table 2**. Among these 20 studies, Most studies demonstrated adequate methodological rigor, particularly in data collection and analysis, although reporting of researcher reflexivity was often limited (Appendices S2).

The included studies covered various prehabilitation approaches: 9 studies (45%) investigated multidimensional prehabilitation programs, including 1 study focusing on tele-prehabilitation; 7 studies examined exercise-based prehabilitation; 1 study evaluated preoperative oral nutrition support (ONS); and 3 studies did not specify the prehabilitation components.

To align with the psychological, social, and behavioral framing introduced in our aim, **Table 2** maps the seven facilitating and four barrier themes onto these three dimensions, along with cross-cutting, organizational, and environmental factors. This mapping does not replace the detailed thematic descriptions below but rather provides a theoretical lens for interpreting the findings.

**Table 2:** Mapping of identified themes onto psychological, social, and behavioral dimensions.

Dimension	Facilitators	Barriers
Psychological	Value recognition	Cognitive and informational barriers; Individual factors
Social	Support systems; Communication strategies	Clinical process limitations; Implementation environment barriers
Behavioral	Motivation and feedback mechanisms; Program design optimization; Process convenience and accessibility	-
Cross-cutting	Introduction mechanisms	-

Psychological, social, and behavioral dimensions represent the core analytical focus of this review. The social dimension is conceptualized broadly to encompass multiple levels—from interpersonal relationships to organizational and environmental contexts. “—” indicates that no factors of this type were identified for the corresponding dimension and category (facilitator or barrier). Cross-cutting, factors are retained to preserve the richness of the original data and to acknowledge the multi-level nature of prehabilitation implementation.

### 3.2 Facilitators of prehabilitation

#### 3.2.1 Introduction mechanisms

The design of introduction mechanisms during prehabilitation implementation demonstrates multifaceted facilitators, with early intervention established as a critical factor. Initiating prehabilitation immediately after diagnosis significantly improves participation rates (39), while early integration into the treatment pathway ensures adequate preparation time. Systematic integration with routine clinical workflows enhances adherence, and a supportive (non-mandatory) inclusion approach improves acceptability (43). Standardized referral pathways further optimize procedural efficiency (40, 44, 46).

Regarding communication strategies, positive interactions during initial assessments help establish trust (43), while clear value communication strengthens engagement motivation (42, 48). Continuous informational support sustains participation willingness (38, 39). Additionally, dynamic screening mechanisms based on clinical indicators, periodic evaluation and adjustment of target populations, and personalized eligibility criteria collectively improve resource allocation (39).

#### 3.2.2 Value recognition

Health benefit-driven motivation emerged as the core facilitator of prehabilitation engagement. This theme, along with others, is mapped onto psychological, social, and behavioral dimensions in **Table 2**. Patients’ primary participation motivation stemmed from perceived health advantages, including improved postoperative self-care capacity, enhanced perioperative physical fitness, overall constitution enhancement, accelerated recovery, and increased survival probability (36–39, 42, 46, 49, 51, 52). These benefit perceptions originated through three channels: (1) clinicians’ professional education thoroughly explaining prehabilitation’s clinical value (39, 44, 49); (2) positive feedback from patients’ prior exercise experiences building trust in interventions (36, 43, 45, 49); and (3) direct experiential gains during prehabilitation, including measurable physical improvement (41, 49). This multidimensional health benefit awareness constituted patients’ intrinsic motivation and a key adherence determinant.

Psychological benefits significantly facilitated prehabilitation participation through multiple mechanisms. Active rehabilitation engagement restored patients’ sense of control over their health, providing crucial psychological support (39, 41). The exercise interventions inherently regulated mood, temporarily alleviating disease focus (36, 38) and effectively diverting attention from negative illness perceptions (40). Notably, some patients developed altruistic dedication from knowing their participation might benefit future patients, achieving self-worth fulfillment (37). During exceptional circumstances (e.g., COVID-19 pandemic), prehabilitation programs additionally served meaningful diversionary purposes for isolated patients (38). These multidimensional psychological benefits collectively strengthened participation persistence and significantly enhanced perceived life quality.

#### 3.2.3 Support systems

Support systems serve as crucial facilitators in prehabilitation implementation, operating through professional and social support dimensions. Professional support is characterized by physiotherapists’ comprehensive supervision ensuring exercise safety and scientific rigor (46, 49), along with multidisciplinary team collaboration that establishes prehabilitation consultation protocols and facilitates hospital-wide engagement to provide systematic rapid referral pathways and comprehensive medical support (39, 40, 44, 46, 52). Social support demonstrates effectiveness through peer strategies enhancing adherence via training exchanges and buddy systems (38, 39, 48, 49), while family involvement not only assists with practical tasks like dietary recording but also generates synergistic effects during program consultations (19, 40, 44, 45, 48, 49, 51, 52). Furthermore, expanded social network participation broadens the support system (19, 40, 49). This integrated support framework combining professional healthcare teams and social relationships provides essential safeguards for effective prehabilitation delivery and serves as a core factor promoting intervention adherence.

#### 3.2.4 Communication strategies

Throughout prehabilitation implementation, patients consistently expressed clear expectations for effective communication strategies spanning the entire intervention process. Patients desired direct, consistent, and reliable communication channels capable of providing timely responses to inquiries and comprehensive ongoing support (19, 38–40, 44, 49–52). Additionally, patients prioritized receiving professional and motivational communication, particularly authoritative recommendations from healthcare providers, which significantly enhanced their confidence in the intervention (39, 40, 44, 45, 49). Patients further demonstrated a preference for concise value explanations that clearly articulated how prehabilitation would concretely improve recovery outcomes (43, 46, 48). When effectively implemented, these communication strategies not only addressed patients’ informational needs throughout prehabilitation but also established trust in clinician-patient relationships, thereby substantially improving participation willingness and intervention adherence.

#### 3.2.5 Motivation and feedback mechanisms

In the implementation of prehabilitation, motivation and feedback mechanisms serve as critical facilitators that play a pivotal role in maintaining patient engagement. Patients commonly reported that immediate progress feedback effectively encouraged sustained participation. This positive reinforcement system encompasses both professional supervision and encouragement from healthcare providers (36, 40, 46, 51, 52) and the sense of achievement gained through self-monitoring—such as satisfaction from completing daily training sessions (37, 38) and self-reflection prompted by analyzing reasons for unmet goals (38). Notably, objective monitoring through digital devices like activity trackers and pedometers (19, 40) not only improved the accuracy of feedback but also enhanced patients’ sense of participation ritual and goal orientation. This multidimensional motivation and feedback system, by integrating external supervision with intrinsic motivation, can significantly improve patient adherence and intervention outcomes.

#### 3.2.6 Program design optimization

In the optimization of prehabilitation program design, core design features can enhance patient participation and adherence. Studies indicate that providing easily understandable instructional information serves as a fundamental facilitator (35, 43–45), requiring prehabilitation programs to have clearly defined goals and content descriptions (39, 48). Well-established program organization and evaluation mechanisms (39), particularly rational scheduling of intervention timelines, ensure systematic feasibility. Furthermore, the availability and accessibility of prehabilitation materials are crucial, with diversified formats (e.g., combining printed and digital materials) (39, 48, 52) better meeting the usage preferences and practical needs of different patient populations. The optimized integration of these design elements collectively forms an essential foundation for effective prehabilitation implementation.

Within prehabilitation program design, personalized adaptation and dynamic adjustment of programs can increase healthcare provider recommendations and patient participation (43). Many patients report that flexible intensity adjustment mechanisms allowing self-reported difficulty levels better align with their practical needs (35, 49). Patients particularly emphasize the importance of designs based on individual baseline conditions, including special considerations for comorbidities (e.g., chronic diseases like diabetes) and specific needs of elderly populations (e.g., frailty status and baseline physical fitness differences) (41, 49–52). They expect exercise plans to be adjusted according to physical condition changes during chemotherapy and training processes (46). Additionally, patients demonstrate clear personal preferences regarding program timing, duration, and training frequency (46), while generally desiring dietary plans that accommodate diverse portion sizes, content, and taste preferences (34, 47). These patient-reported perspectives suggest that systematic integration of personalized design elements may help prehabilitation programs better adapt to diverse population needs (43).

#### 3.2.7 Process convenience and program accessibility

During prehabilitation implementation, process convenience and program accessibility were identified by patients as significant facilitators. Patients generally expressed a preference for local training participation, with prioritized community-based healthcare facility placement and convenient program locations significantly reducing engagement barriers (35, 38, 39, 44, 48, 51, 52). The integration of digital tools not only decreased patients’ commuting requirements but also enhanced clinician-patient interaction, with this flexible approach better aligning with contemporary patient lifestyles (19, 39, 40). Furthermore, low-cost program designs and dedicated prehabilitation time allocation (35, 36, 38–41, 44, 46, 49, 50) helped eliminate additional participation obstacles, thereby improving program accessibility and sustainability. The comprehensive optimization of these elements reflects patients’ practical needs for convenient, flexible, and economically feasible prehabilitation services.

### 3.3 Barriers of prehabilitation

#### 3.3.1 Cognitive and informational barriers

Cognitive and informational barriers significantly hinder prehabilitation participation during implementation. A prominent issue is healthcare professionals’ insufficient awareness of prehabilitation importance, with some clinicians maintaining skeptical attitudes (39, 52), resulting in inadequate patient education about prehabilitation benefits and even instances where “doctors forget preoperative explanations” (43). Patients also demonstrate substantial knowledge gaps, as many lack sufficient understanding of prehabilitation advantages due to inadequate clinician counseling, with some believing “routine postoperative care suffices” (43). This perception may stem from certain procedures’ inherently low complication rates (e.g., colorectal surgery) (39) or personal biases that prehabilitation “helps others but not themselves” (47). Deeper cognitive barriers include traditional beliefs such as “requiring rest after cancer diagnosis” or “prioritizing immediate surgery” (39, 44, 49, 51). Furthermore, current educational materials’ design flaws—emphasizing content descriptions over value explanations (43, 46, 48)—limit effective information dissemination. These multilayered cognitive and informational barriers collectively compromise prehabilitation acceptance and participation rates.

#### 3.3.2 Individual factors

Individual factors constitute multi-layered barriers to prehabilitation participation. Physical limitations include chemotherapy side effects (e.g., appetite loss and physical discomfort) that significantly impair dietary and exercise adherence (47), while pre-existing comorbidities (e.g., diabetes) and poor baseline physical capacity (including ambulatory impairments) further increase participation challenges (38, 49). Psychological barriers demonstrate greater complexity, with patients experiencing emotional distress and high-pressure states post-diagnosis (40, 42, 49, 51), often exhibiting medical care dependency and lacking proactive engagement (40). Additional psychological obstacles include: low exercise confidence (fear of incorrect movements or inexperience) (43, 49, 51); excessive risk perception regarding physical activity (45, 49); cognitive biases like “self-perceived adequate fitness” and guilt over missed sessions (even worrying incomplete tasks may increase preoperative stress) (43, 50); concerns about dietary impacts on gastrointestinal function; safety apprehensions regarding home-based exercise (particularly elderly patients’ fear of falling) (45, 50); and privacy concerns regarding activity tracker usage (47, 48). The interplay of these physiological, psychological, and social factors collectively influences patients’ prehabilitation acceptance and adherence.

#### 3.3.3 Clinical process limitations

Clinical process limitations constitute systemic barriers to prehabilitation implementation. Time constraints emerge as the most prominent challenge, where short intervals between diagnosis and surgery (including unexpected schedule advancements) compress prehabilitation periods (35, 36, 39–41, 44, 46, 49, 52), with some patients actively shortening prehabilitation to avoid surgical delays (49). Preoperative logistical burdens (e.g., frequent visitor interactions) and competing priorities further divert patients’ attention and time resources (44, 47), while time-intensive consultation processes exacerbate scheduling pressures (39). Extended waiting times for psychological support were also noted (51). Referral system deficiencies—including lacking rapid access channels and multidisciplinary coordination inefficiencies (e.g., resolving specialty scheduling conflicts)—hinder seamless prehabilitation initiation (39, 44). More complexly, surgical scheduling conflicts with prehabilitation timelines (39, 44, 49, 50, 54) reveal divergent perspectives: clinicians (particularly surgeons) endorse safely delaying surgery for prehabilitation (39, 44), whereas patients and informal caregivers often resist this approach (44, 49). This cognitive discrepancy introduces additional uncertainty in prehabilitation scheduling. These interacting clinical process barriers collectively constrain effective prehabilitation delivery.

#### 3.3.4 Implementation environment barriers

Inadequate resource allocation constitutes a significant systemic barrier to prehabilitation implementation. Insufficient healthcare system support, particularly regarding specialized staff shortages (e.g., hospital-based physiotherapists) (39, 46, 52), directly compromises intervention quality, with participants reporting difficulties sustaining programs due to incorrect or misunderstood exercise guidance (46). Institutional-level challenges, including misalignment with hospital-wide strategic priorities and difficulties implementing new programs (39, 40), reflect systemic support deficiencies. Physical space constraints (e.g., limited capacity), inadequate exercise facilities (40, 50), and excessive specialist workloads due to absent multidisciplinary teams (40, 46) further hinder effective delivery.

Resource accessibility presents multiple obstacles. Long travel distances deter participation (38, 39, 44, 49). Employment-related scheduling conflicts (39) similarly constrained participation by limiting patients’ available time to attend prehabilitation sessions. Insufficient family support (e.g., conflicting dietary preferences) reduces adherence motivation (44, 47, 49), and limited digital access impedes remote prehabilitation adoption (40). Financial burdens—including out-of-pocket costs from non-reimbursed programs (35, 39, 44, 52) and perceived burdens from excessive compliance measurements (e.g., pre-/post-meal weighing, photo documentation, weekly food diaries) (49)—further restrict engagement. Uncontrollable factors like severe weather also disrupt programming (41, 46). These interconnected resource-level barriers collectively undermine prehabilitation accessibility and sustainability.

## 4 Discussion

This qualitative systematic review synthesized 20 studies to identify the barriers and facilitators to prehabilitation participation among adult cancer patients awaiting surgery. Our analysis revealed seven facilitating factors and four barrier factors, which were mapped onto psychological, social, behavioral, cross-cutting dimensions, with the social dimension encompassing both interpersonal and structural/organizational factors. The findings demonstrate that prehabilitation engagement is shaped by a complex interplay of individual-level psychological states (e.g., value recognition, fear, self-efficacy), social processes (e.g., family support, peer encouragement, communication strategies, and structural conditions such as service accessibility and financial resources), and behavioral mechanisms (e.g., feedback loops, self-monitoring, program flexibility). Critically, our synthesis reveals a”cognitive-behavioral gap” —many patients recognize the value of prehabilitation yet encounter barriers that prevent them from translating this recognition into sustained action—highlighting the need for interventions that go beyond education to address modifiable psychological, social, and behavioral determinants.

### 4.1 Comparison with existing qualitative syntheses

Our findings both align with and extend previous qualitative syntheses in this field. Scriney et al. (29), published in Psycho-Oncology, examined the impact of prehabilitation on affective and functional outcomes in young to midlife adult cancer patients. While their review focused on intervention effects, our review addresses the determinants of participation—a complementary but distinct question. Together, these two reviews suggest that prehabilitation is not only effective but also acceptable to patients, yet participation remains suboptimal due to modifiable barriers.

van der Velde et al. (22) and Watts et al. (23) conducted mixed-methods systematic reviews on barriers and facilitators to prehabilitation. Both identified similar factors (e.g., time constraints, social support, patient knowledge). However, their reliance on quantitative survey data limited their ability to capture the experiential richness and contextual nuance that qualitative methods uniquely provide. Our review fills this gap by synthesizing in-depth patient and provider narratives, revealing how fear manifests as specific concerns about incorrect movements, how guilt over missed sessions creates additional preoperative stress, and how traditional beliefs about rest persist despite clinical recommendations.

We view our review as complementary to these existing syntheses—offering an inductive, patient-centered perspective with explicit focus on psychological, social, and behavioral dimensions as modifiable intervention targets.

### 4.2 A psychological, social, and behavioral interpretation of the findings

Our findings can be interpreted through an integrative framework that positions psychological, social, and behavioral dimensions as interacting determinants of prehabilitation engagement.

#### 4.2.1 Psychological dimension

Consistent with health behavior theories such as the COM-B model (26), participants’ motivation to engage was primarily driven by perceived health benefits—improved recovery, enhanced physical fitness, and increased survival probability. This “value recognition” acted as a psychological catalyst, with some patients reporting that their diagnosis motivated them to engage in prehabilitation (51). However, a persistent “cognitive-behavioral gap” emerged: many patients knew prehabilitation was beneficial but did not act on this knowledge. This gap was maintained by psychological barriers including fear of incorrect movements, low exercise confidence, illness-related distress, and deeply held traditional beliefs such as “rest is needed after cancer diagnosis” or “surgery should not be delayed.” These findings suggest that addressing cognitive biases and enhancing self-efficacy are prerequisite steps before behavioral change can occur.

#### 4.2.2 Social dimension

Social factors operated at multiple levels. At the interpersonal level, family involvement, particularly from partners, provided practical and moral support that sustained participation (49, 51). Peer support through buddy systems and training exchanges normalized the prehabilitation experience and reduced feelings of isolation (38, 48). Healthcare professionals’ encouragement—especially when delivered through clear, authoritative, and personalized communication—significantly enhanced patients’ confidence and trust (39, 40). At the structural level, organizational and environmental conditions—including service accessibility, financial resources, and employment policies—shaped the availability and quality of these interpersonal supports. These two levels interacted: patients who faced travel distance, employment conflicts, or financial burden often could not attend sessions with family members or peer groups, thereby missing opportunities for the very social support that might have sustained their engagement (39, 44, 49).

#### 4.2.3 Behavioral dimension

Behavioral mechanisms—feedback, self-monitoring, and program flexibility—emerged as critical levers for translating psychological motivation and social support into sustained action. Immediate progress feedback, whether from wearable devices or clinician encouragement, reinforced participation by providing tangible evidence of improvement (19, 40). Self-monitoring, such as completing daily training logs or using pedometers, transformed abstract goals into concrete, achievable tasks (37, 38). Flexible program design—allowing patients to adjust intensity, timing, and modality based on their physical condition and personal preferences—accommodated the heterogeneity of cancer patients, including those with comorbidities, frailty, or ongoing chemotherapy (35, 46, 49). These behavioral features are not add-ons but core components that determine whether psychological readiness translates into actual participation.

#### 4.2.4 Cross-dimension interactions

Our synthesis also revealed that the three dimensions do not operate in isolation. For example, value recognition (psychological) is most effective when reinforced by family encouragement (social) and immediate feedback (behavioral). Conversely, time constraints (organizational) exacerbate psychological distress, which in turn undermines behavioral adherence. Understanding these interactions is essential for designing integrated interventions.

### 4.3 In-depth discussion of three core themes

Among the identified themes, three warrant deeper discussion because they span multiple dimensions, have direct clinical implications, and represent the most frequently cited barriers or facilitators across the 20 included studies.

#### 4.3.1 Theme 1: time constraints—the most pervasive barrier

Time constraints emerged as the most frequently cited barrier across studies. Short intervals between diagnosis and surgery (often 1–3 days) compress prehabilitation periods (35, 36, 39–41, 44, 46, 49). Importantly, time constraints operate across multiple dimensions: they create psychological pressure (patients feel rushed and anxious), generate cognitive conflict (clinicians endorse delaying surgery for prehabilitation while patients resist), and produce structural barriers, as short time windows between diagnosis and surgery—shaped by both institutional scheduling priorities (39) and patients’ concerns about delaying cancer treatment (44)—limited the time available for prehabilitation. This multidimensional impact suggests that addressing time constraints requires not only scheduling adjustments but also shared decision-making to align patient and clinician expectations, as well as flexible or intensive prehabilitation protocols that can be completed in shorter time windows (46, 49).

#### 4.3.2 Theme 2: cognitive and informational barriers—the “invisible” obstacles

A striking finding was that barriers are not only practical but also cognitive and informational. Many patients held traditional beliefs that directly contradict prehabilitation principles (e.g., “rest is needed after cancer diagnosis,” “surgery should not be delayed”), while others believed prehabilitation “helps others but not themselves” (47). Equally concerning, healthcare professionals themselves demonstrated insufficient awareness of prehabilitation importance, with some clinicians maintaining skeptical attitudes or “forgetting preoperative explanations” (39, 43). This reveals a failure of knowledge translation—even when evidence exists, it has not effectively changed clinical beliefs and practices. Educational materials compounded the problem by emphasizing what prehabilitation is rather than why it matters (43, 46, 48). Addressing these invisible barriers requires value-based education for both patients and providers, emphasizing specific, quantifiable benefits rather than program descriptions, while acknowledging evidence uncertainties to avoid overpromising (20).

#### 4.3.3 Theme 3: support systems and communication—the most powerful facilitators

Across all 20 studies, support systems consistently emerged as the most powerful facilitators of prehabilitation engagement. Family involvement provided not only practical assistance(e.g., dietary recording, transportation) but also moral support that sustained motivation during difficult periods (19, 40, 44, 45, 48, 49). Peer support through buddy systems offered experiential validation and reduced isolation (38, 39, 48, 49). Professional support—particularly from physiotherapists who provided comprehensive supervision and from multidisciplinary teams that established rapid referral pathways—ensured both safety and scientific rigor (39, 40, 44, 46, 49). Notably, Saggu et al. (51) introduced a novel concept of “life preparation”(e.g., will-making, financial planning, household arrangements) among ovarian cancer patients, suggesting that prehabilitation programs may need to address practical life concerns that extend beyond traditional physical and psychological domains, particularly for patients with advanced disease.

### 4.4 Implications for clinical practice, program design, and policy

Our findings translate into actionable strategies across three levels.

#### 4.4.1 Clinical practice

Prehabilitation programs should incorporate structured psychological assessment before participation to identify patients with low self-efficacy, fear, or cognitive barriers. Value-based education—clearly articulating specific, quantifiable benefits—should complement or support content-focused descriptions, yet must balance encouragement with honest acknowledgment of evidence uncertainties to support informed decision-making (20). Overemphasizing benefits may create unrealistic expectations, while acknowledging limitations can foster trust. Our findings indicate that such communication should address both cognitive barriers (e.g., unclear explanations) and structural barriers (e.g., time, cost, travel), without placing the onus solely on individual motivation. Systematic integration of family support and multidisciplinary team coordination is essential for sustained engagement.

#### 4.4.2 Program design

Prehabilitation programs must be flexible and personalized. Key design features include: graded goal-setting (starting with small, achievable tasks), immediate feedback mechanisms (activity trackers, daily logs, regular check-ins), multiple delivery modalities (facility-based, home-based, remote/tele-prehabilitation), and accommodations for specific populations (older adults, patients with comorbidities, those undergoing neoadjuvant therapy). Behavioral reinforcement strategies—such as self-monitoring tools and progress tracking—should be embedded as core components, not optional add-ons.

#### 4.4.3 Policy and health systems

Healthcare systems must address structural barriers that individual-level interventions cannot overcome. This includes: establishing standardized referral pathways, allocating dedicated prehabilitation funding and staffing (particularly physiotherapists and prehabilitation nurses), providing physical space and equipment, and considering reimbursement mechanisms to reduce out-of-pocket costs. Prehabilitation should be considered a quality indicator for cancer surgery programs, similar to ERAS compliance metrics.

### 4.5 Future research directions

Our findings suggest a three-step implementation framework “Identify—Communicate—Design” to guide clinical practice: (1) Identify individual barriers and facilitators through structured psychosocial assessment, enabling tailored support for patients with low self-efficacy, fear, or limited social resources; (2) Communicate value-based, risk-honest information with family involvement, balancing encouragement with realistic expectations to support informed decision-making; and (3) Design flexible, graded, and accessible programs that proactively address structural barriers such as time, travel, and cost, without placing the onus solely on individual motivation.

Our findings also point to several directions for future research. First, mixed-methods studies should investigate the cognitive-behavioral gap—why patients who recognize the value of prehabilitation remain unable to participate—and examine how services can be redesigned to reduce structural inequalities. Second, multi-stakeholder qualitative research, incorporating perspectives from clinicians, administrators, and policymakers, is needed to identify systemic barriers beyond the patient level. Third, longitudinal research would clarify how barriers evolve over time, and cross-cultural research would establish whether the factors identified here generalize across different healthcare systems. Fourth, implementation science approaches—such as intervention mapping or CFIR—should translate these findings into testable programs that can be evaluated in pragmatic trials.

## 5 Limitations

This study has several limitations. First, the evidence base is predominantly patient-focused, which is appropriate for understanding patient-level barriers and facilitators. While the inclusion of a professional perspective (52) adds valuable frontline insights, it is not sufficient to fully capture systemic and institutional barriers. Second, some data were collected postoperatively, introducing potential recall bias, although this design allows for comprehensive evaluation of the entire prehabilitation experience. Third, our analysis employed descriptive thematic synthesis rather than more interpretative approaches such as constructivist grounded theory, which might have generated deeper theoretical insights into how and why factors interact. Fourth, we restricted inclusion to English and Chinese language publications, potentially omitting relevant studies from other linguistic contexts. Fifth, although we conducted an updated search through May 31, 2025, the rapid growth of this field means new evidence may have emerged since our search date. Sixth, the heterogeneity in prehabilitation definitions, content, and duration across included studies limits the comparability of findings. Seventh, we did not search grey literature (e.g., dissertations, conference proceedings, preprints), which may have introduced publication bias. However, the inclusion of 20 studies from diverse journals and countries partially mitigates this concern.

## 6 Conclusions

Preoperative prehabilitation for cancer patients involves complex, multidimensional barriers and facilitators that span psychological, social, behavioral, organizational, and environmental domains. Our synthesis reveals a persistent “cognitive-behavioral gap”: patients recognize the value of prehabilitation yet encounter psychological, social, and behavioral barriers that prevent sustained engagement. Addressing this gap requires integrated, multi-level strategies that: (1) enhance self-efficacy and address cognitive biases (psychological); (2) mobilize family, peer, and professional support (social); (3) embed feedback, self-monitoring, and flexible design features (behavioral); and (4) address time constraints, referral pathways, and resource shortages (organizational/environmental). By explicitly mapping barriers and facilitators onto psychological, social, and behavioral dimensions, this review provides a conceptual framework that can guide both intervention design and future research, moving beyond a “one-size-fits-all” approach toward truly patient-centered prehabilitation. Such comprehensive, patient-centered approaches are essential to optimize prehabilitation participation and ultimately improve surgical outcomes for cancer patients.

## Data Availability

The original contributions presented in the study are included in the article/**Supplementary material**. Further inquiries can be directed to the corresponding author.
