# Supplementary material for: Psychological, social, and behavioral barriers and facilitators to prehabilitation before cancer surgery: a qualitative systematic review
Source: Front Oncol. 2026 Sep 17;16:1903631. doi: 10.3389/fonc.2026.1903631 (PMC13627339; doi:10.3389/fonc.2026.1903631)
Supplement: Supplementary file 1 [file Table1.docx]

**Supplement 1. Barriers and facilitators of prehabilitation before cancer surgery: A qualitative systematic review –Initial search conducted on January 13, 2025**

This appendix presents the search strategies for the initial search conducted on January 13, 2025. The updated search (May 31, 2025) used the same strategies and identified two additional eligible studies.

| **Database** | **Search Term and Search Query** | **Numbers** |
| --- | --- | --- |
| CNKI | 篇名：预康复 + 术前康复 + 术前优化 + 术前干预 + 术前活动 + 术前运动 + 术前预康复+ 术前AND摘要：质性研究 + 现象学 + 民族志 + 访谈法 + 焦点小组+ 扎根理论 + 行动研究 + 半结构访谈 + 混合性研究 | 71 |
| WanFang Data | 题名：预康复 OR 术前康复 OR 术前优化 OR 术前干预 OR 术前活动 OR 术前运动 OR 术前预康复AND 题名或关键词：质性研究 OR 现象学 OR 民族志 OR 访谈法 OR 焦点小组OR 扎根理论 OR 行动研究 OR 半结构访谈 OR 混合性研究 | 173 |
| VIP | 预康复 OR 术前康复 OR 术前优化 OR 术前干预 OR 术前活动 OR 术前运动 OR 术前预康复OR 术前 AND 质性研究 OR 现象学 OR 民族志 OR 访谈法 OR 焦点小组OR 扎根理论 OR 行动研究 OR 半结构访谈 OR 混合性研究 | 87 |
| Sinomed | [(( "质性研究"[摘要:智能] OR "现象学"[摘要:智能] OR "民族志"[摘要:智能] OR "访谈法"[摘要:智能] OR "焦点小组OR 扎根理论"[摘要:智能] OR "行动研究"[摘要:智能] OR "半结构访谈"[摘要:智能] OR "混合性研究"[摘要:智能])) AND (( "预康复"[标题:智能] OR "术前康复"[标题:智能] OR "术前优化"[标题:智能] OR "术前干预"[标题:智能] OR "术前活动"[标题:智能] OR "术前运动"[标题:智能] OR "术前预康复"[标题:智能] OR "术前"[标题:智能]))](javascript:toDoRelimitSearch();) | 68 |
| PubMed | (“prehabilitation”[Title/Abstract] OR “preoperative exercise”[MeSH Terms] OR “prehabilitat*”[Title/Abstract] OR “pre-operative exercise”[Title/Abstract] OR “preoperative rehabilitation” [Title/Abstract] OR “preoperative physical therapy”[Title/Abstract] OR “preoperative intervention”[Title/Abstract] OR “preoperative care”[Title/Abstract]) OR “post-operative rehabilitation” AND  ((((((((Qualitative Research[Title/Abstract]) OR (Phenomenology[Title/Abstract])) OR (Ethnography[Title/Abstract])) OR (face-to-face Interview*[Title/Abstract])) OR (Focus Group[Title/Abstract])) OR (Grounded Theory[Title/Abstract])) OR (Semi-structured Interview*[Title/Abstract])) OR (Mixed Methods Research[Title/Abstract])) OR (Mixed Methods Study[Title/Abstract]) OR (qualitative study[Title/Abstract])) | 124 |
| Web  of Science | #1 ((((((((TI=(prehabilitation)) OR TI=(preoperative exercise)) OR TI=(prehabilitat*)) OR TI=(pre-operative exercise)) OR TI=(preoperative rehabilitation)) OR TI=(preoperative physical therapy)) OR TI=(preoperative intervention)) OR TI=(preoperative care)) OR TI=(post-operative rehabilitation)  #2 (((((((((TI=(Qualitative Research)) OR TI=(Phenomenology)) OR TI=(Ethnography)) OR TI=(face-to-face Interview*)) OR TI=(Focus Group)) OR TI=(Grounded Theory)) OR TI=(Semi-structured Interview*)) OR TI=(Mixed Methods Research)) OR TI=(Mixed Methods Study)) OR TI=(qualitative study)  #3 (((((((((AB=(Qualitative Research)) OR AB=(Phenomenology)) OR AB=(Ethnography)) OR AB=(face-to-face Interview*)) OR AB=(Focus Group)) OR AB=(Grounded Theory)) OR AB=(Semi-structured Interview*)) OR AB=(Mixed Methods Research)) OR AB=(Mixed Methods Study)) OR AB=(qualitative study)  #1 AND(#2 OR#3) | 241 |
| The Cochrane Library | Title Abstract Keyword：prehabilitation or preoperative exercise or prehabilitat* or pre-operative exercise or preoperative rehabilitation or preoperative physical therapy or preoperative intervention or preoperative care or post-operative rehabilitation  Title Abstract Keyword：qualitative research or phenomenology or ethnography or face-to-face Interview* or focus group or grounded theory or semi-structured interview* or mixed methods research or mixed methods study or qualitative study | 43 |
| Embase | #1 prehabilitation:ab,ti OR 'preoperative exercise':ab,ti OR prehabilitat*:ab,ti OR 'pre-operative exercise':ab,ti OR 'preoperative rehabilitation':ab,ti OR 'preoperative physical therapy':ab,ti OR 'preoperative intervention':ab,ti OR 'preoperative care':ab,ti OR 'post-operative rehabilitation':ab,ti  #2'qualitative research':ab,ti OR phenomenology:ab,ti OR ethnography:ab,ti OR 'face-to-face interview*':ab,ti OR 'focus group':ab,ti OR 'grounded theory':ab,ti OR 'semi-structured interview*':ab,ti OR 'mixed methods research':ab,ti OR 'mixed methods study':ab,ti OR 'qualitative study':ab,ti  #1 AND #2 | 142 |
| CINAHL  **（CINAHL Plus with Full Text）** | #1 TI：prehabilitation OR preoperative exercise OR prehabilitat* OR pre-operative exercise OR preoperative rehabilitation OR preoperative physical therapy OR preoperative intervention OR preoperative care OR post-operative rehabilitation  #2 AB：prehabilitation OR preoperative exercise OR prehabilitat* OR pre-operative exercise OR preoperative rehabilitation OR preoperative physical therapy OR preoperative intervention OR preoperative care OR post-operative rehabilitation  #3 AB ( Qualitative Research OR Phenomenology OR Ethnography OR face-to-face Interview* OR Focus Group OR Grounded Theory OR Semi-structured Interview* OR Mixed Methods Research OR Mixed Methods Study OR qualitative study) OR TI ( Qualitative Research OR Phenomenology OR Ethnography OR face-to-face Interview* OR Focus Group OR Grounded Theory OR Semi-structured Interview* OR Mixed Methods Research OR Mixed Methods Study OR qualitative study)  (#1 OR #2) AND #3 | 233 |
| **Total** | / | 1182 |
