# Supplementary material for: Psychological, social, and behavioral barriers and facilitators to prehabilitation before cancer surgery: a qualitative systematic review
Source: Front Oncol. 2026 Sep 17;16:1903631. doi: 10.3389/fonc.2026.1903631 (PMC13627339; doi:10.3389/fonc.2026.1903631)
Supplement: Supplementary file 2 [file Table2.docx]

Table 2 Main outcome of included studies

| Author(Year) | Main outcome |
| --- | --- |
| Hogan et al., 2019 | Major themes associated with compliance included flavour, volume, texture, impact on dietary intake and motivation to consume supplements. |
| Agasi-Idenburg et al., 2020 | 1) Limited time, not receiving or misunderstanding information, physical ailments, and emotional impact of the diagnosis were identified as barriers to preoperative exercise. 2) Not having physical complaints , understandable information provided by a physician, and low-cost programs were reported as facilitate exercise. 3) Participants suggested that exercise should not be too intensive and should be adjusted to personal preferences and should be provided close to home. |
| Karlsson et al., 2020 | 1) Support from others emerged as an important possibility for overcoming the barriers. 2) When physical exercise was considered a natural part of life, and when it had previously resulted in positive feedback, patients expressed an openness towards engaging in preoperative physical exercise. 3) Environmental factors—including physical, social, attitudinal, and psychological aspects—were described as influencing preoperative physical exercise in either inhibiting or facilitating way. 4) Competing priorities were also identified, with some patients reporting a different focus of interest than physical activity during the preoperative period. 5) Participants described prerequisites for initiating physical exercise, either independently or with support from others, including active support, tools to perform the exercise, encouragement and monitoring from healthcare professionals, guidance, structure, accessibility and time set aside for exercise. 6) The strongest motivation for pursuing preoperative physical activity was the goal of achieving postoperative independence. 7) A lack of communication from healthcare professionals regarding preoperative physical exercise was reported as a barrier. |
| Beck et al., 2020 | 1) A strong motivational factor identified was the belief that participation in a research study served a higher purpose (helping future patients). 2) In relation to the actions performed, the patients found it motivating that they were to write down what they had completed in relation to prehabilitation on a daily basis. 3) Last but not least, the patients hoped and believed that prehabilitation would strengthen their bodies, leading to early discharge, which motivated them to act. Also, simply the act of doing something made the patients feel calm, meaning that it was not always the ultimate goal of the act (e.g., early discharge) that motivated them, but the feeling of calmness that taking action gave rise to. |
| Cooper et al., 2022 | 1) Patients reported that taking part in ChemoFit could increase their chance of surviving the operation and recovering thereafter. 2) They expressed a sense of obligation to the clinical team to improve their fitness for surgery. 3) Tracking progress provided an incentive to continue with the intervention. 4) Maintaining activity levels while undergoing chemotherapy was reported as challenging. 5) Strength exercises were difficult to master and maintain. 6) Barriers to walking made achieving step targets difficult. 7) Exercising at home helped overcome barriers including time and travel. 8) Cardiopulmonary exercise testing on a static bike proved challenging. 9) Completing the exercise programme was a welcome distraction from their illness. 10) Having someone present when the intervention was described was considered beneficial. 11) Ongoing support from the clinical team was reported as vital for maintaining motivation and adherence. 12) Self-monitoring of physical activity levels—including reasons for not achieving goals—was considered beneficial. 13) Peer support was identified as potentially beneficial for adherence. 14) ChemoFit provided a means of doing something meaningful during the COVID-19 pandemic lockdown. |
| Heil et al., 2022 | Barriers:   1) Combining appointments was reported as difficult due to different work activities. 2) Contradictory and low-quality scientific evidence for (cost-)effectiveness was identified. 3) Costs needed to be financed immediately while yields were not directly clear. 4) Counseling patients was reported as time-consuming. 5) Differences in patients' resilience and training opportunities were noted. 6) Effectiveness was considered difficult to prove due to heterogeneity of the patient population. 7) The goal and content of the prehabilitation program were reported as unclear. 8) Healthcare professionals were reported as unaware of the importance of prehabilitation. 9) The healthcare system was not adapted, including availability of paramedics in hospitals. 10) Indirect costs for patients (e.g., travel expenses) were identified. 11) Lack of program organization and evaluation was reported. 12) Multidisciplinary consultation was reported as time-consuming. 13) Operating room planning took precedence over the prehabilitation program. 14) Patients were unable to visit the hospital frequently. 15) Quality of care for colorectal surgery was already high with low complication rates. 16) There was uncertainty about which group benefited most from prehabilitation. 17) The belief that sedentary behavior was necessary after cancer diagnosis was reported. 18) The belief that the tumor should be removed as soon as possible was identified. 19) The time between operation indication and surgery was reported as too short.   Facilitators：   1) Accessible contact between involved healthcare professionals was reported as a facilitator. 2) Adjusting patient selection during implementation based on local results was suggested. 3) Having an ambassador to persuade, enthuse, and unite coworkers was identified. 4) Application of prehabilitation fitting within hospital strategy was reported. 5) Awareness regarding the impact of surgery on physical condition was noted. 6) Both objective and patient-reported outcomes were considered important for program evaluation. 7) Combining appointments on a single day was reported as a facilitator. 8) Coordination of the program and appointments by a specialized nurse was identified. 9) Delaying surgery if necessary was reported as feasible. 10) Evaluation of individual patients only in case of signaled problems or deviation from the program was suggested. 11) Evidence regarding effectiveness of prehabilitation was considered important for program sustainability. 12) Group activities to exchange experiences and motivate peers were reported. 13) Guaranteeing financial support was identified. 14) Incorporating the social environment to facilitate patient engagement was suggested. 15) Implementation of digital tools for interaction and reduction of travel distance was reported. 16) Including skeptical healthcare professionals in the prehabilitation team from the adoption phase was suggested. 17) Individualized programs were reported as important. 18) Insight into movement patterns was noted. 19) Introducing prehabilitation as part of regular care was identified. 20) Introducing prehabilitation early in the trajectory was reported. 21) Offering an intervention program close to home was identified as a facilitator. 22) Patients being able to improve their self-reliance instead of just waiting was reported. 23) Personal support during the prehabilitation program was identified. 24) Preoperative multidisciplinary prehabilitation consultation was reported. 25) Setting goals and motivating patients to accomplish them was identified. |
| van der Zanden et al., 2022 | Motivation  Motivational reasons: 1) Patients reported feeling urgency; 2) Patients felt they could contribute to their own recovery; 3) Prehabilitation served as a distraction from negative thoughts.  Motivational support:  Individual: 1) Individualized approaches; 2) Seeing good results; 3) Setting goals; 4) Use of activity trackers/pedometers.  Support of others: 1) Supervision and motivation by a professional; 2) Support of family and friends; 3) Specialist involvement in prehabilitation; 4) Doing exercises together with others.; 5) "Big stick" (like going somewhere)  Practical facilitators: 1) Prehabilitation as part of routine care; 2) A motivated team.  Practical barriers  Practical barriers for patients: 1) Short time period between diagnosis and operation; 2) Going to hospital for prehabilitation; 3) Too intensive a program; 4) Referral to another hospital  Practical barriers in organization: 1) Financial objections; 2) Capacity of space / personnel; 3) Much asked of gynecologists; 4) Poor communication  between healthcare professionals; 5) Lack of knowledge or motivation among gynecologists; 6) Difficulty implementing something new; 7) Lack of an evidence based program.  Patient related factors: 1) Stressful period for patients; 2) Physical condition of patient; 3) Lack of knowledge among patients; 4) Lack of motivation among patients; 5) Fixed patterns or habits of patients; 6) Patient dependent of care; 7) Limited access to digital resources; 8) Language barriers; 9) The confronting nature of the program. |
| Wu et al., 2022 | 1) They described the interactions with members of the prehabilitation team as key facilitators in their ability to participate. 2) A prominent theme highlighted during the interviews was the convenience of teleprehabilitation. 3) A key opportunity patients felt was missing from tele-prehabilitation was the chance to share advice with other patients. 4) In acute settings, all the patients believed that participation in the service would enable them to withstand the adverse consequences of their cancer treatment, leading to better health outcomes and quicker recovery. 5) The patients agreed that the support from their family and friends served as a strong motivator of their engagement in the prehabilitation programme. 6) Patients said the availability of support from the prehabilitation team helped to track their progress and maintain engagement. 7) Many patients had a positive mindset toward their health outcomes and disease. They perceived this positive mindset as a motivation to engage with our intervention programme and to cope with their disease and recovery. |
| Barnes et al., 2023 | Facilitators included: 1) The program being manageable and suitable for older adults with frailty；2) Adequate resources to support engagement,；3) Support from others；4) A sense of control, intrinsic value, noticing progress and improving health outcomes；5) The program being enjoyable and facilitated by previous experience.  Barriers included: 1) Pre‐existing conditions, fatigue and baseline fitness；2) Weather；3) Guilt and frustration when unable to exercise. A need for individualization and variety was offered as a suggestion by participants and was therefore described as both a barrier and a facilitator. |
| Paulo et al., 2023 | Five relevant themes of barriers to PA prehabilitation were identified: 1) Mental factors；2) Personal responsibilities；3) Physical capacity； 4) Health conditions；5) Lack of exercise facilities.  Facilitators contributing to PA prehabilitation adherence in kidney cancer included：1) Holistic health；2) Social and professional support；3) Acknowledgment of health benefits； 4) Exercise type and guidance；5) Communication channels. |
| Powell et al., 2023 | 1) Preoperative demands were reported as excessive, with prehabilitation adding to the burden. 2) 2) Prehabilitation introduction strategies were considered important, with brief explanations improving acceptance. 3) Doctors' failure to introduce prehabilitation was reported to reduce patient acceptance. 4) A short time to surgery could affect whether or not patients could be referred. 5) Having surgery brought forward made it impossible for patients to attend their planned assessment with programme staff. 6) Balancing cancer treatment and prehabilitation involved differing perspectives between doctors and patients. 7) The possibility that taking part in prehabilitation could improve recovery seemed to be a major motivating factor for patients. 8) Previous own or vicarious experience seemed to influence both non-engagers’perceptions that standard post-operative care would suffice. 9) Patients' insufficient understanding of the importance of prehabilitation was reported as leading to refusal. 10) Treating prehabilitation as part of routine care seemed to be perceived as increasing adherence. 11) Treating prehabilitation as part of routine care, without carefully discussing it with patients and considering their views, could be counterproductive. 12) A lack of pressure within programme delivery was seen as beneficial, with a supportive rather than directive approach being appreciated. 13) Engagement also seemed to be supported by general positive experiences with programme staff at initial assessment. 14) Greater awareness of the tailoring of exercises to suit individuals’levels and abilities could potentially encourage healthcare staff to recommend the programme, and encourage patients to participate. 15) Being ‘already physically fit or active’ was selected by some as a perceived reason for patients declining referral to the P4C Programme. 16) Being unfamiliar with exercise was seen as a factor that could deter participation. 17) From a ‘clinician’ perspective, exercise experience seemed to be a factor that could impact participation. 18) Patients' exercise experience pre-diagnosis could affect likelihood of participation.19) Some ‘clinician’ participants endorsed ‘lack of confidence in exercising’ as a reason for declining referral. 20) Many ‘clinicians’ endorsed ‘a buddy strategy’ as a way to help patients to take part in prehabilitation. |
| Voorn et al., 2023 | 1) According to all healthcare professionals, patients should be informed on how they can positively influence their health and functioning preoperatively. 2) Healthcare professionals mentioned the short period between diagnosis and surgery as a barrier for prehabilitation, which might be too short to initiate an effective prehabilitation program. 3) Most healthcare professionals reported that surgery could be delayed safely when the pulmonologist or surgeon decides that a delay of surgery is possible. 4) They mentioned that referring patients to prehabilitation might be facilitated when multiple professionals within and between primary and secondary healthcare were involved in prehabilitation and have weekly time slots available to schedule patients quickly. 5) Most healthcare professionals prefered prehabilitation to take place at the hospital because communication between healthcare professionals within the hospital was easier. 6) Healthcare professionals encouraged informal caregivers to be involved in prehabilitation as they could stimulate their loved ones and know how to motivate them. 7) Patients having to pay the costs associated with preoperative preventive interventions themselves was reported as a barrier. 8) When the patients received information about prehabilitation, they believed it facilitated postoperative recovery and mentioned that they would participate in prehabilitation if their physician recommended it. 9) Some patients stated that they had a busy preoperative period because friends and family visited them at home, which was experienced as a barrier to participating in prehabilitation. 10) The opportunity of having a direct communication with a healthcare professional was mentioned as a facilitator for patients to take part in a prehabilitation program. 11) Patients preferred a physical exercise training program organized in primary care, because it fits better within their living context. 12) Group-based exercises or having a training buddy were preferred by most patients, because of contact with other patients and motivational reasons. 13) A long travel distance was seen as a barrier to participating in prehabilitation. 14) Unsupervised prehabilitation at home was seen as a barrier for most patients, as they mentioned a lack of self-discipline. 15) Patients felt they needed the support of their loved ones and preferred that they could join the appointments in a prehabilitation program. 16) Divergent attitudes toward delaying surgery for prehabilitation were reported. 17) Informal caregivers preferred prehabilitation for their loved ones to be organized in their own living context in primary care in order to provide optimal support. 18) Most informal caregivers reported that they wanted the tumor to be resected at the earliest convenience and did not prefer a delay of surgery in favor of prehabilitation. |
| Wang et al., 2023 | 1) Physical capacity to tolerate exercise intensity; 2) Psychological concerns about exercise risks; 3) Adequate informational support and professional guidance; 4) Simple and feasible exercise prescriptions and rehabilitation conditions; 5) Intrinsic motivation from recognizing the value of prehabilitation ; 6) Positive influence of existing exercise habits; 7) Sustained motivation from perceived exercise benefits; 8) Dual encouragement from group dynamics and family support. |
| Casanovas-alvarez et al., 2024 | 1) Patients acknowledged that being aware of that information might have had increased their motivation to participate in the study. 2) They also reported that the greatest motivation for joining the program was to be guided and supervised by a physiotherapist specializing in oncology and to share those difficult moments by exercising outdoors (outside the hospital grounds) with people in the same situation where they could share and exchange experiences. 3) Patients also acknowledged that although medical prescription was the oncologist’s first and most important role, they should also include the discussion of other topics such as the benefits of exercise to manage side effects from treatment, as this would enhance the motivation for participation. 4) All participants began to report feeling both the physical and emotional benefits of the exercise supervised by a physiotherapist. 5) The type of supervision provided by the physiotherapist and the organization of the sessions were aspects that participants reported as positive reasons for continuing with the sessions. 6) Barriers identified included: (i) side effects from chemotherapy; (ii) lack of information and medical referral to prehabilitation; and (iii) adverse weather conditions. Facilitators identified included: (i) professionalism and follow-up by the physiotherapist; (ii) safety of supervised exercise; (iii) individualization of exercises according to patient status; (iv) accessibility of facilities, frequency, and timing; and (v) mutual encouragement from group members. 7) Exercises were adapted to each participant according to their fluctuating conditions during chemotherapy. 8) The physical, psychological, and social benefits experienced by participants were reported as the main facilitators for adhering to the program. 9) Accessibility of facilities was also reported as important. 10) Having sufficient time was identified as another important factor. 11) The most valued facilitator was group support and the cohesion participants felt among themselves. 12) Easy access to outdoor facilities and the hospital surroundings were seen as facilitators of the prehabilitation program. 13) Materials used during prehabilitation were reported. 14)Participants did not feel safe exercising without professional guidance, adaptation, and supervision, particularly when they still had numerous sequelae. 15) Lack of Guidance by the multidisciplinary team was identified as a barrier. 16) Insufficient Information on side effects was also reported as a barrier. |
| Clemons et al., 2024 | 1) Poorly timed recruitment efforts; 2) Misconceptions about prehabilitation diet recommendations; 3) Competing priorities that made prehabilitation less feasible; 4) Lack of family alignment; 5) Belief that prehabilitation would not be helpful; 6) Concerns over specific prehabilitation program components; and 7) Belief that prehabilitation is helpful for others but not for themselves; 8) Discordant family food preferences made dietary recommendations difficult to follow; 9) Patients had concerns over specific prehabilitation program components. For example, participants who had been on chemotherapy felt that they would not want to adopt a particular diet because they wanted to have the full range of options available to them, as chemotherapy affected their appetites. Patients undergoing gastrointestinal surgery were concerned about adopting any new diet that might affect their digestive system. One patient also cited privacy concerns because the prehabilitation program described to him involved wearing an activity tracker. |
| Eser et al., 2024 | 1) Prehabilitation Location; 2) Training Methods (with/without companions [peer and self], digita and paper-based training); 3) Training Duration; 4) Involvement of Family or Peers (differing perspectives); 5) Need for a Designated Contact Person for addressing diagnostic, surgical, and hospitalization queries; 6)Concerns About Wearable Device Data Security/Privacy; 7) Critiques of Prehabilitation Program flyer design (must clearly communicate benefits rather than just content); 8) Long Travel Distances as a Participation Barrier for hospital-based sessions, though some patient-professional interaction was considered desirable. |
| Sier et al., 2024 | 1）Logistical problems and the impact of cancer diagnosis were most frequently reported as barriers to participation. 2) Patients preferred obtaining the information on paper as well. 3) They expressed the wish to bring an extra person with them to the consultation. 4) The information provided was clear, hence most patients did not see any additional value in an informational video. 5) Some patients opted for a shortened prehabilitation period to avoid delaying their scheduled surgery. 6) Patients were most motivated by the expected beneficial impact of prehabilitation on preoperative physical fitness and postoperative recovery. 7) This program offered them the opportunity to play a role in their own recovery. 8) This made them receptive to such an offer, especially when it was recommended by their healthcare professional. 9) The ignorance of what would happen during treatment motivated the participants to take advantage of all available opportunities to help them get through it in the best possible way. 10) Other factors that stimulated participation were the support of family, the fact that the program was free, having sufficient time to participate due to retirement, and previous experience with on-site rehabilitation. 11) Travelling to the hospital three times a week, in addition to other hospital appointments, was reported as challenging. 12) One patient perceived his comorbidity as a barrier. 13) All patients preferred an in-hospital exercise program, because of the expertise of the staff and the facilities available. 14) Patients indicated that they had no confidence in a home-based exercise program due to lack of supervision and the anticipation of constantly postponing exercises. 15) Patients with prior experience of community physiotherapy preferred in-hospital training, as they perceived community physiotherapists as having less time, attention, and expertise. 16) Three sessions per week were sufficient for all patients. 17) All patients preferred training on weekdays, in the morning or afternoon. 18) According to patients, the content of the exercise program—a circuit of strength and high-intensity exercises—could remain the same. 19) Some patients experienced the intensity as heavy in the beginning, but overall, it was manageable. 20) Patients reported the importance of the patients indicating themselves when exercises were too hard, as too demanding exercises could lead to non-compliance. 21) Patients should be instructed to speak up because some might be afraid to do so. 22) Transportation issues were identified as a barrier. 23) The number of patients per physiotherapis was reported as a concern. 24) Peer support was identified as beneficial. 25) Timely feedback for progress motivation was considered important. Most patients expressed that compliance could best be measured by the physiotherapists. 26) Some patients appreciated the efforts made but did not like the content and taste of the meals; these patients did not consume the full meal with all required nutrition. 27) The compliance measurements (weighing dishes before and after consumption, taking photos of meals, and keeping a food diary for one week) were experienced as an extra burden. 28) Oversized portions were identified as a barrier.29) Patients suggested improving adherence by allowing them to specify preferences regarding meal content and quantity. 30) Patients recommended that medical conditions (such as diabetes) should be taken into account in meal composition. 31) Family support emerged as the most important form of support, followed by support from acquaintances, friends, religion, medical experts, and fellow patients. 32) Two patients would have preferred a conversation or extra time with their healthcare professional instead of a session with a psychologist. 33) Some patients reported no need for psychological support. 34) Others reported feeling more confident and better prepared for surgery after prehabilitation. 35) Several participants stated that prehabilitation provided mental distraction and a sense of structure. |
| Zhang et al., 2024 | 1）Some patients viewed preoperative prehabilitation merely as a clinical task assigned by healthcare providers, participating despite lacking genuine belief in its efficacy. 2) A minority rejected prehabilitation's value, fearing that undertaking but failing to complete the program might increase preoperative psychological stress. 3) Institutional constraints (inadequate activity space in hospitals) and home environment limitations (lack of professional equipment) were reported as significant participation barriers. 4) Safety concerns emerged as the paramount consideration for elderly early-stage lung cancer patients in home-based prehabilitation, with fall risks and preoperative symptoms such as cough differentially impacting adherence. 5) The unpredictable preoperative period—affected by variable hospital bed scheduling and patients' decision timelines—frequently compromised prehabilitation implementation. 6) Elderly patients strongly preferred simplified exercise regimens with timely feedback, necessitating age-adapted protocols tailored to physical capabilities and exercise preferences. 7) These patients sought communication with optimistic peers who had undergone similar surgeries, valuing this for psychological preparation. 8) They additionally requested designated clinical contacts for sustained communication during the home-based preoperative waiting period. |
| Saggu et al., 2025 | Facilitators: 1) Positive mindset as a coping mechanism (staying positive, viewing surgery as a curative opportunity); 2) Diagnosis as a "teachable moment" motivating lifestyle changes; 3) Self-directed preparation (physical activity, nutrition, psychological practices); 4) Support from family (especially partners for domestic and moral support) and healthcare teams (sufficient time for humane conversation); 5) Faith as an important support system; 6) Remote delivery preferred by patients with heavy hospital burden or long travel distances; 7) Group sessions valued for motivation (though some avoided them to prevent social comparison).  Barriers: 1) Tumor-related comorbidities (ascites, fatigue) limiting physical activity; 2) Lack of clear explanation about prehabilitation pathways; 3) Long waiting times for psychological support; 4) Cultural taboos around gynaecological cancers limiting open discussion; 5) Concerns about remote delivery (face-to-face preferred by some for better assessment); 6) Unmet need for psychological support due to resource constraints; 7) "Life preparation" (wills, finances, family arrangements) taking priority over prehabilitation. |
| Zhang et al., 2025 | Facilitators: 1) Recognised importance of prehabilitation for improving surgical tolerance and reducing complications; 2) Clinical professionals' positive attitudes and willingness to implement; 3) Leadership support from hospital administration; 4) Most patients willing to accept prehabilitation; 5) Initial ERAS-MDT framework providing conditions for implementation.  Barriers: 1) Lack of clinical practice knowledge (confusion between prehabilitation and preoperative preparation); 2) Insufficient preoperative preparation time (1-3 days before surgery); 3) Lack of aging-friendly clinical practice schemes (exercise not tailored to elderly preferences, materials not age-adapted); 4) Immature multidisciplinary cooperation mechanism (unclear division of labour, lack of incentives); 5) Lack of explicit regulations for monitoring and supervision; 6) Lack of emergency safeguards for home-based prehabilitation; 7) Shortage of specialised professionals (e.g., lung cancer specialist nurses).  Practice strategies: Development of evidence summaries, healthcare training materials, anpatient health education brochures; clarification of ERAS-MDT division of labour; improvement of patient safety and monitoring measures; optimisation of practice flow; obtaining funding support. |
